# Endothelial GABA signaling: a phoenix awakened

**DOI:** 10.18632/aging.101457

**Published:** 2018-05-22

**Authors:** Yong Kee Choi, Anju Vasudevan

**Affiliations:** 1 Department of Psychiatry, Harvard Medical School, Boston, MA 02215, USA; 2 Angiogenesis and Brain Development Laboratory, Division of Basic Neuroscience, McLean Hospital, Belmont, MA 02478, USA

**Keywords:** GABA, endothelial cells, angiogenesis, brain development, psychiatric disease, autism, epilepsy, schizophrenia

Abnormalities in GABA signaling in the forebrain at all stages - prenatal, postnatal and adult have been implicated with a wide range of neuropsychiatric illnesses [1,2]. Additionally, age related decline of GABA concentrations in the cerebral cortex has been associated with poor cognition and neurodegenerative diseases [3]. Thus, GABA’s versatility in functions at different stages of life in different brain regions is vital to its significance. As the first transmitter to become functional in the embryonic brain, GABA shapes cortical networks, and itself facilitates the developmental switch from excitatory in the prenatal brain to inhibitory in the postnatal brain [4]. Elegant and extensive studies have shown us the importance of the multifaceted actions of GABA. GABA has sometimes been referred to as a ‘phoenix’ that cyclically regenerates, renewing excitement and interest, time after time. In this regard, recent studies in our laboratory unfolds a new GABA signaling pathway in the prenatal brain, that is distinct from the traditional GABA signaling pathway described hitherto in neurons [5]. It highlights the importance of a new cell type specific source that secretes GABA in the embryonic forebrain - the endothelial cells and establishes novel autonomous links between blood vessels and the origin of neuropsychiatric diseases like autism, epilepsy and schizophrenia.

Though there have been reports of GAD immunoreactivities in endothelial cells of cerebral arteries [6] and GAD65 expression in cardiac arteries and major blood vessels of embryos [7], its functional significance was not known. Several mouse models with abnormal GABA_A_ receptors and GABA function with defective behaviors have been vital for understanding the pathobiology of neuropsychiatric illnesses, but all of these models were systemic or region-specific knockouts that could not serve to establish a cause - effect relationship between neuronal versus endothelial cells. Our observation of expression of GABA_A_ receptor subunits and GAD65/67 in forebrain endothelial cells signified that forebrain angiogenesis has its own intrinsic GABA signaling mechanisms. To discover the functional significance of this endothelial GABA signaling pathway *in vivo*, we designed strategies to specifically turn off GABA release from endothelial cells or render endothelial GABA_A_ receptors dysfunctional using cre-lox technology during early embryonic development. Both approaches first affected forebrain angiogenesis and in turn impaired neuronal migration. As endothelial GABA’s roles were delineated individually for angiogenesis, neurogenesis, long distance GABAergic neuronal migration and radial migration of projection neurons, so too was the realization that neuronal GABA could not serve to compensate these roles. The data laid the foundation for a novel positive feedback signaling pathway in endothelial cells that functions via GABA_A_ receptor mediated GABA release [5]. Concurrent vascular and GABA cell deficits persisted in the adult brain and significantly impacted postnatal behavior, similar to neuropsychiatric diseases that are characterized by one or more of these core symptoms - impaired social interactions, communication deficits, depression, increased anxiety and seizures. In addition, the results provided novel evidence that variations in endothelial GABA levels during prenatal brain development can contribute to diversity in psychiatric disease symptoms [5].

**Figure 1:**
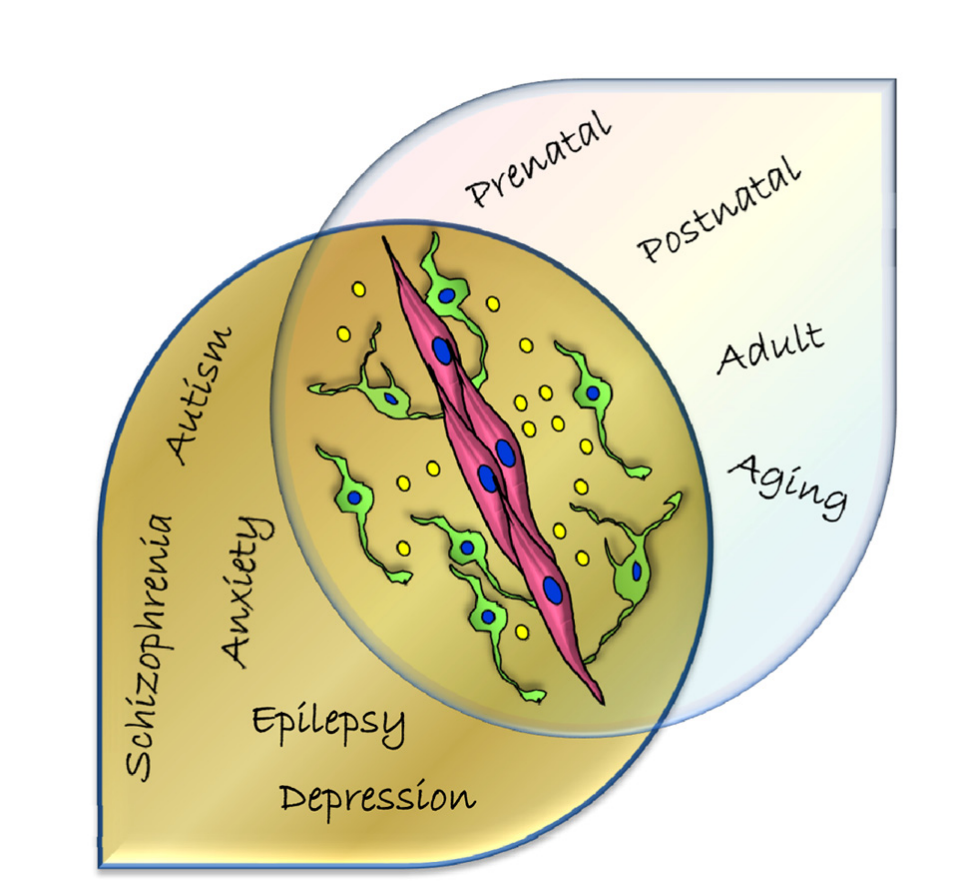
Schema illustrating the significance of endothelial GABA mediated signaling for neurovascular interactions and its implications for a wide range of neurological and psychiatric diseases. In addition to the prenatal brain, it is important to gain further insights into mechanisms and functions of this novel GABA pathway in the postnatal, adult and aging brain. Depictions: Endothelial cells (pink), GABAergic neurons (green), GABA (yellow circles).

As we now know that a common GABA pathway operates in both endothelial cells and GABAergic neurons of the embryonic telencephalon, it is essential to gain further mechanistic insights by segregating this pathway in individual cell types. It is important for future work to understand how the endothelial GABA signaling pathway specifically influences angiogenesis related genes and specific processes like tight junction formation, tip cell functions, vascular sprouting and migration. Also, it is important to see how components of the neuronal GABA pathway are affected in the absence of endothelial GABA signaling. For instance, how does endothelial GABA modulate interneuron migration - does it affect receptor-mediated signaling that transduces extracellular information or directly the transcription factors that provide interneurons with their intrinsic migratory ability or both? It also brings up several new questions with respect to roles of GABA_A_ receptors, NKCC1-KCC2 expression, and chloride concentration in forebrain endothelial cells and alterations in disease scenarios. And most importantly, additional questions begin to emerge about the significance of this vascular GABA pathway and novel mechanisms specifically in the postnatal, adult and aging brain (Figure 1). New understanding of endothelial GABA’s temporal and chronological role is of major importance since the vasculature is an effective target for tranquilizers, sedatives, anaesthetics and psychoactive medications at all stages of life.
